# APRI and FIB-4 for Liver Fibrosis Assessment in Chronic Hepatitis B: Evidence from a Hyperendemic Andean Setting

**DOI:** 10.3390/medsci14060670

**Published:** 2026-10-13

**Authors:** Carol Celeste Cordero Vargas, Evy Yamil Huaman Huallanca, Homero Ango-Aguilar, Obert Marín-Sánchez, Ruy D. Chacón, Jimmy H. Ango-Bedriñana

**Affiliations:** 1 Escuela Profesional de Medicina Humana, Facultad de Ciencias de la Salud, Universidad Nacional de San Cristóbal de Huamanga, Ayacucho 05001, Peru; corderovargascarol50@gmail.com (C.C.C.V.); evyyamilhuaman@gmail.com (E.Y.H.H.); 2 Programa Académico de Microbiología, Facultad de Ciencias Biológicas, Universidad Nacional de San Cristóbal de Huamanga, Ayacucho 05001, Peru; homero.ango@unsch.edu.pe; 3 Departamento Académico de Microbiología Médica, Facultad de Medicina, Universidad Nacional Mayor de San Marcos, Lima 09001, Peru; omarins@unmsm.edu.pe; 4 Pathogen Genetics Research Group (PATHO-GEN), Organization for Medical Innovation and Collaboration for Sciences (OMICS), Lima 15072, Peru; ruychaconv@alumni.usp.br

**Keywords:** chronic hepatitis B, liver fibrosis, transient elastography, aspartate aminotransferase-to-platelet ratio index, APRI, fibrosis-4 index, FIB-4, non-invasive tests, diagnostic agreement, Peru

## Abstract

**Background/Objectives:** The 2024 WHO guidelines incorporated the APRI index into treatment decisions for chronic hepatitis B, with thresholds of >0.5 for significant fibrosis and >1.0 for cirrhosis, because it can be computed when neither biopsy nor elastography is available. The evidence supporting those thresholds derives almost entirely from studies using liver biopsy as reference standard; how serological indices behave when the available comparator is elastography remains unknown, and no data exist for Andean populations. We aimed to quantify the agreement of APRI and FIB-4 with transient elastography, determine whether either discriminates better, and evaluate the 2024 WHO thresholds in this setting. **Methods**: Retrospective agreement study of patients in the Hepatitis B Elimination Plan of Huanta, Peru. APRI and FIB-4, computed from routine laboratory results, were compared against transient elastography (FibroScan 402, M+ probe) used as a reference standard given the unavailability of biopsy. Outcomes followed the 2024 WHO thresholds: >7.0 kPa for significant fibrosis and >12.5 kPa for cirrhosis. Only examinations meeting the pre-specified quality criterion (at least 10 valid measurements; IQR/median ≤ 30%) were analyzed. Diagnostic properties were estimated with exact confidence intervals and likelihood ratios, areas under the curve with bootstrap intervals, and the between-index comparison with DeLong’s test for paired curves. Reporting followed STARD 2015. **Results**: Of 92 patients, 73 had a valid elastography examination; APRI was computable in 60 and FIB-4 in 57. Prevalence was 23.3% (95% CI 14.2–34.6) for significant fibrosis and 12.3% (5.8–22.1) for cirrhosis. For significant fibrosis, APRI > 0.5 achieved 61.5% sensitivity (31.6–86.1) and 74.5% specificity (59.7–86.1), with a positive likelihood ratio of 2.41 (1.26–4.62) and a negative one of 0.52 (0.25–1.05). For cirrhosis, APRI > 1.0 yielded 71.4% sensitivity, 92.5% specificity and a positive likelihood ratio of 9.46 (3.30–27.11). Areas under the curve were 0.844 and 0.808 for significant fibrosis and 0.914 and 0.874 for cirrhosis; DeLong’s test detected no statistically significant difference between indices at either level (*p* = 0.47 and 0.53); however, the limited number of outcome-positive cases (13 and 7, respectively) means that clinically important differences cannot be excluded and equivalence has not been established. **Conclusions**: APRI and FIB-4 identified cirrhosis with likelihood ratios large enough to act on but were only weakly informative at the threshold that governs treatment eligibility, where the negative likelihood ratios did not exclude unity. Neither index can be used as a stand-alone rule-out test at that threshold. The small number of outcome-positive cases limits precision throughout. Although the program intended both assessments to occur during the same session, individual dates were unavailable, preventing verification of the actual interval between elastography and blood sampling.

## 1. Introduction

Chronic hepatitis B virus (HBV) infection can progress to liver fibrosis, cirrhosis, and hepatocellular carcinoma. Accurate assessment of liver fibrosis is therefore an important component of chronic HBV care because the identification of significant fibrosis or cirrhosis influences treatment eligibility and clinical surveillance. However, access to fibrosis staging remains limited in many low- and middle-income countries (LMICs) and in rural settings, where specialized diagnostic technologies may not be routinely available.

Liver biopsy has historically been considered the reference standard for fibrosis staging, but its invasive nature, cost, sampling variability, and need for specialized expertise limit its use in routine clinical practice. Transient elastography provides a non-invasive assessment of liver stiffness and is widely used for fibrosis staging, but its implementation depends on access to specialized equipment and trained personnel. These limitations are particularly relevant in resource-limited settings, where the World Health Organization (WHO) recommends the use of non-invasive tests rather than liver biopsy for fibrosis assessment [1,2]. The 2024 WHO hepatitis B guidelines specifically recommend the aspartate aminotransferase-to-platelet ratio index (APRI) as a preferred non-invasive test in resource-limited settings, while transient elastography may be preferable when available and affordable [2].

Among simple blood-based fibrosis indices, APRI and the fibrosis-4 index (FIB-4) are particularly attractive because they can be calculated from routinely available clinical laboratory measurements. APRI combines aspartate aminotransferase (AST) and platelet count and was originally developed as a simple non-invasive marker of significant fibrosis and cirrhosis [1]. FIB-4 combines age, AST, alanine aminotransferase (ALT), and platelet count and was originally developed by Sterling et al. (2006) in patients with HIV/HCV co-infection [3]. Thus, both indices require no specialized equipment and can be readily calculated in peripheral healthcare facilities. Their simplicity and low resource requirements have contributed to their incorporation into non-invasive fibrosis assessment strategies, particularly in settings where elastography is not readily accessible [2,4].

For chronic HBV infection, accumulated evidence supports the potential utility of these indices, although their diagnostic performance varies according to the population, disease spectrum, threshold, and reference standard used. A large systematic review and meta-analysis including 211 studies and 61,665 patients reported moderate diagnostic performance of APRI for significant fibrosis, with substantially different sensitivity and specificity depending on the selected threshold [4]. The 2024 WHO guidelines consequently recommend an APRI threshold of >0.5 as evidence of significant fibrosis (≥F2), or a transient elastography of >7.0 kPa, and an APRI threshold of >1.0 or transient elastography of >12.5 kPa, together with clinical criteria, as evidence suggestive of cirrhosis (F4) [2]. Nevertheless, these recommendations are intended to facilitate treatment decisions at the population and health-system level, and the performance of APRI and FIB-4 may vary when applied to specific populations and clinical settings [5].

Two issues are particularly relevant to their use in underserved populations. First, much of the evidence supporting non-invasive fibrosis indices has relied on liver biopsy as the reference standard, whereas in routine practice, transient elastography may represent the most feasible objective comparator when it is available. Diagnostic agreement with elastography may therefore differ from performance estimates obtained against histological staging. Studies conducted in resource-limited settings have reported substantially lower sensitivity of APRI when transient elastography is used as the reference method [6,7]. Second, evidence remains limited for several populations in Latin America and other underrepresented regions, including Andean populations. Previous research on HBV in Peru has also been concentrated in Lima-based institutions, with limited representation of departments where HBV has historically been highly endemic [8]. Moreover, viral genotype may also contribute to differences in the clinical course and treatment response of chronic hepatitis B, with genotype F being particularly relevant in indigenous and admixed populations of the Americas [9].

Ayacucho represents an important setting in this context. The province of Huanta was historically hyperendemic for HBV, with an HBsAg prevalence of approximately 20% reported in 1985–1986. Although childhood vaccination substantially reduced HBV transmission, subsequent resurgence has been documented, and Ayacucho has remained among the Peruvian regions with high reported incidence [10]. Consequently, a population of people living with chronic HBV requires access to fibrosis assessment within a healthcare network where specialized staging technologies are not routinely available. During implementation of the Hepatitis B Elimination Plan, transient elastography became temporarily available at a rural referral hospital in Huanta, providing an unusual opportunity to evaluate the performance of simple blood-based indices against an objective non-invasive fibrosis assessment in the population in which they may ultimately be used.

Metabolic dysfunction-associated steatotic liver disease (MASLD) is an important public health concern in Latin America [11]. Concurrent hepatic steatosis may also affect the performance of non-invasive fibrosis indices, including APRI and FIB-4 [12,13]. Therefore, this study aimed to evaluate the diagnostic performance and agreement of APRI and FIB-4 for identifying significant liver fibrosis and cirrhosis, using transient elastography as the available non-invasive reference method, among adults with chronic hepatitis B in Huanta, Peru.

## 2. Materials and Methods

### 2.1. Design, Setting and Participants

This was a retrospective observational agreement study based on a review of the clinical records of patients attended within the Hepatitis B Elimination Plan of Huanta province, Ayacucho, Peru, funded by the Ministry of Health. Data were derived from records generated during routine care, not for research purposes. The study was conducted at the Hospital de Apoyo de Huanta, a provincial referral facility located at 2642 m above sea level (12°56′19″ S, 74°14′55″ W), between June and December 2019.

Patients aged 18 years or older with an established diagnosis of chronic hepatitis B who underwent transient elastography, viral load, complete blood count with platelets and transaminases were included.

Patients were excluded if a valid and reliable liver stiffness measurement could not be obtained. An examination was considered technically valid when at least ten valid measurements were acquired with acceptable variability between them, defined as an interquartile range to median ratio (IQR/M) of 30% or less. Examinations yielding seven to nine valid measurements were released with observations and counted only towards the technical success rate; they were not analyzed. Examinations with fewer than seven valid measurements were not released. Because the device available had no XL probe, patients with obesity in whom anthropometric characteristics prevented a valid and reliable measurement with the M probe were likewise excluded. Co-infection with hepatitis C or HIV, liver disease of other etiology, hazardous alcohol use and ongoing antiviral treatment were not applied as exclusion criteria and are not consistently identifiable in the clinical record; their potential influence is addressed in the Limitations.

Sampling was consecutive and non-probabilistic: all patients in the period meeting the criteria were included. No a priori sample size calculation was performed; sample size was determined by the duration of the Plan’s intervention component. Under the Plan’s protocol, the initial evaluation was intended to take place in a single session, with transient elastography, blood sampling and the initial medical assessment performed on the same day; the planned interval between the index tests and the reference standard was therefore zero days. The protocol did not specify a maximum permissible interval because both assessments were intended to occur during the same session. The clinical review of results took place approximately one month later and did not involve repeat testing. However, the individual dates of elastography and blood collection were not recorded, so adherence to the intended timing could not be verified for each participant.

The study was conducted in accordance with the ethical principles of the Declaration of Helsinki and was reviewed and approved by the Research and Ethics Committee of the Hospital Regional de Ayacucho, an institutional committee constituted by Directorial Resolution No. 033-2014-GRA/GG-GRDS-DRSA/HR”MAMLL”A-DE (Certificate of Approval No. 015-2021, 2 March 2021).

### 2.2. Index Tests: APRI and FIB-4

Both indices were computed from laboratory results obtained during routine care at the Hospital de Apoyo de Huanta.

APRI = [(AST ÷ ULN_AST) × 100] ÷ platelets (10^9^/L)

FIB-4 = [age (years) × AST] ÷ [platelets (10^9^/L) × √ALT]

The upper limit of normal for AST was 40 U/L. This value was verified by reconstruction from the AST, platelet count and index stored in the source database: the resulting quotient was exactly 40 in all 75 patients with a computable APRI, with zero standard deviation.

AST and ALT were expressed in U/L and platelet count in 10^9^/L. Cut-offs were pre-specified from the literature: 0.5 and 1.5 for APRI, and 1.45 and 3.25 for FIB-4. The 1.0 threshold for APRI, recommended by WHO in 2024 for cirrhosis, was additionally evaluated [4]. No data-derived cut-offs are proposed: threshold optimization was removed from the analysis because, with 13 outcome-positive cases, it would give such cut-offs more prominence than the data support.

Under the Plan’s intervention protocol, transient elastography was performed at the point of patient enrolment and before laboratory results were available, in order to make use of the contact with the patient. At the time of performing and interpreting the examination, the operator therefore had no access to AST, ALT or platelet values and could not know the APRI or FIB-4 results, which were computed subsequently from the laboratory output. The reference standard was thus obtained independently of, and prior to, the index tests (STARD items 10 and 13) [14].

### 2.3. Reference Standard and Why It Is Not a Gold Standard

Elastography was performed with a FibroScan 402 device (Echosens, Paris, France) fitted with a 3.5 MHz M+ probe, by a single operator trained in transient elastography with six months of experience. A minimum fast of six hours was required, together with ten valid measurements and an IQR/M ratio of 30% or less; the technical success rate was 90%. During the study period, 490 examinations were carried out, of which 5.4% were not released under the pre-specified quality criteria set out in Section 2.1.

Outcomes were defined using the thresholds recommended in the 2024 WHO guidelines: a liver stiffness above 7.0 kPa for significant fibrosis (≥F2) and above 12.5 kPa for cirrhosis (F4) [2]. We adopted these rather than the operational cut-offs applied during the Plan (6.9, 9.4 and 12 kPa) because the latter are not traceable to a documented validation in chronic hepatitis B, whereas the WHO values are the reference against which treatment eligibility is now decided. The two sets are closely aligned in this cohort: reclassification affected a single patient, whose stiffness was exactly 6.9 kPa, and no patient fell between 12.0 and 12.5 kPa; all estimates obtained with the original thresholds are reported as a sensitivity analysis. Advanced fibrosis (≥F3) is no longer a primary outcome and is retained only as a secondary analysis, since the elastographic threshold for that stage is the least consistently defined of the three. It should be noted that WHO itself states that transient elastography “has no validated exact cut-off for specific fibrosis stages” and presents 7 and 12.5 kPa as mean values usable for treatment decisions [2]. The published cut-offs for chronic hepatitis B support that statement, and their variability is both between studies and within them. In a prospective multicenter study of 173 patients with biopsy as reference, the optimal values were 7.2 kPa for significant fibrosis and 11.0 kPa for cirrhosis when the sum of sensitivity and specificity was maximized, but 7.2 and 18.2 kPa when diagnostic accuracy was maximized instead—the same data yielding cirrhosis thresholds that differ by 7 kPa according to the optimization criterion alone [15]. Across studies, the dispersion is comparable: a meta-analysis of 18 studies and 2772 patients estimated 7.9 kPa for significant fibrosis across a range of 6.1 to 11.8 kPa, 8.8 kPa for advanced fibrosis across 8.1 to 9.7 kPa, and 11.7 kPa for cirrhosis across 7.3 to 17.5 kPa [16]. The operational threshold of 9.4 kPa used during the Plan for advanced fibrosis lies between the two values reported for that stage in the prospective study (8.1 and 10.5 kPa) and within the range reported by the meta-analysis, which supports its clinical plausibility without establishing a documented validation.

Liver biopsy was unavailable at the facility and across the provincial network. Elastography was therefore used as the reference standard; it is accepted as the best-performing direct non-invasive method but is itself subject to misclassification, particularly in the presence of steatosis, marked necroinflammatory activity, cholestasis, or thoracoabdominal adiposity. Consequently, the estimates reported here describe the agreement of the indices with elastography, not their accuracy against true histological status. Because the errors of the index tests and of the reference standard may be conditionally dependent—steatosis and necroinflammatory activity affect liver stiffness and the transaminases alike—misclassification by the reference standard may bias sensitivity, specificity and agreement in different directions. We therefore make no claim about the direction of that bias and report our estimates as agreement with transient elastography rather than as bounds on accuracy against histological fibrosis. This caveat governs the interpretation of the entire study.

The order of the two tests is fixed by the Plan’s protocol, elastography preceding the laboratory work-up, but the database does not record the dates of either, so the length of the interval between index test and reference standard could not be estimated (STARD item 22) [14].

### 2.4. Other Variables

Sex, age, body mass index (BMI) and HBV viral load by real-time quantitative PCR were recorded. Body mass index was of interest because of its association with technical failure of M-mode elastography. The original protocol also included Child-Pugh and MELD; neither could be computed because prothrombin time and INR were unavailable, and both were excluded.

### 2.5. Statistical Analysis

Analysis was by complete cases, without imputation. A STARD 2015 flow diagram [14] was constructed, and baseline characteristics of included versus excluded patients were compared to assess selection bias.

Continuous variables are described with median and interquartile range, categorical variables with absolute and relative frequencies. For each index and cut-off, 2 × 2 tables were constructed against each fibrosis level, and sensitivity, specificity, predictive values and likelihood ratios were estimated with exact Clopper–Pearson 95% confidence intervals. Discrimination was assessed by the area under the ROC curve, with intervals from 2000 bootstrap replicates. The comparison between APRI and FIB-4 used DeLong’s test for correlated curves, restricted to the subset with both indices and elastography available so that the comparison was paired. A two-sided *p*-value below 0.05 was considered significant. Hepatitis B viral load was measured in international units per milliliter (IU/mL); undetectable results were kept distinct from quantified values and were not imputed. An exploratory stratified analysis evaluated the sensitivity of FIB-4 above 1.45 for significant fibrosis in patients aged ≤35 and >35 years. Spearman’s rank correlation was used to assess the associations of age and liver stiffness with FIB-4.

Analyses were performed in R 4.6.0 with the pROC 1.19 and psych packages. Results were independently reproduced in a different computing environment (R 4.3.3, pROC 1.18), agreeing to the fourth decimal place across all estimates, including bootstrap intervals. Analysis code is available (Supplementary File S1). Reporting follows the STARD 2015 checklist [14].

## 3. Results

### 3.1. Participant Flow and Characteristics

Of the 92 eligible patients, 85 had a recorded transient elastography examination, of whom 73 met the pre-specified quality criterion. Eleven examinations had an IQR/M ratio above 30%, and one had no recorded IQR/M ratio. Among the 73 patients meeting the quality criterion, APRI was computable in 60 and FIB-4 in 57; the paired set for the comparison of both indices comprised 57 patients. Missing index values were due to unavailable platelet counts (12 patients), AST (4) and ALT (7); these categories may overlap. Complete-case sets comprised 70 patients for APRI–elastography and 67 for FIB-4–elastography and for the paired comparison of both indices (Figure 1).

Included and excluded patients did not differ in age (median 34 versus 37 years; *p* = 0.80), body mass index (23.8 versus 22.3; *p* = 0.20) or sex (*p* = 0.16).

Median age was 36 years (IQR 28–46; range 19–78), with an exactly balanced sex distribution (46 men; 46 women). Median body mass index was 23.4 kg/m^2^ (IQR 20.3–27.0); a total of 15 patients (16.3%) exceeded 28 kg/m^2^, the threshold above which M-mode elastography failure increases (Table 1). Of the 92 patients, 85 had a recorded elastography examination, and 73 met the quality criterion of Section 2.1 (11 exceeded an IQR/M of 30%, and 1 had no IQR/M recorded). Within those 73, APRI was computable in 60 and FIB-4 in 57; the paired set used for the between-index comparison comprised 57 patients (Figure 1). Missing index values were due to absent platelet counts (12), AST (4) and ALT (7).

### 3.2. Fibrosis Distribution

Among the 73 patients with a valid elastography examination, median liver stiffness was 4.9 kPa (IQR 4.0–6.8; range 2.4–70.6). Seventeen had significant fibrosis (23.3%; 95% CI 14.2–34.6), and nine had cirrhosis (12.3%; 5.8–22.1) (Table 2).

### 3.3. Diagnostic Properties

Diagnostic properties for each index and cut-off at the predefined fibrosis thresholds are shown in Table 3.

The pattern differs between the two levels. For significant fibrosis, both indices are moderately specific but insensitive, and their positive likelihood ratios, although above unity, are small. For cirrhosis, specificity exceeds 92%, and the positive likelihood ratios are an order of magnitude larger, while negative predictive values exceed 96%.

### 3.4. Discrimination and Between-Index Comparison

Areas under the curve and the paired between-index comparison are given in Table 4 and displayed in Figure 2.

No statistically significant difference between APRI and FIB-4 was detected at either level. With 13 cases of significant fibrosis and 7 of cirrhosis, this does not establish equivalence, and clinically important differences cannot be excluded.

Spearman correlations with liver stiffness were ρ = 0.572 for APRI and ρ = 0.547 for FIB-4 (both *p* < 0.001); between indices, ρ = 0.752. BMI showed a weak, non-significant correlation with liver stiffness (Spearman’s ρ = 0.205; *p* = 0.082).

### 3.5. Viral Load and Sensitivity Analysis

As a secondary analysis at the advanced-fibrosis level, retained with the original 9.4 kPa threshold, FIB-4 above 1.45 reached a sensitivity of 66.7% and a specificity of 79.2% (9 cases). In an exploratory age-stratified analysis, the sensitivity of FIB-4 above 1.45 for significant fibrosis was 25.0% among patients aged ≤35 years (4 cases) and 66.7% among those aged >35 years (9 cases). Age correlated strongly with FIB-4 (Spearman’s ρ = 0.729; *p* < 0.0001) but only modestly with liver stiffness (ρ = 0.306). These findings should be interpreted cautiously because of the small number of cases in each age stratum.

Viral load showed weak inverse correlations with liver stiffness (ρ = −0.240; *p* = 0.065) and APRI (ρ = −0.293; *p* = 0.041). Median viral load was lower in patients with significant fibrosis than in those without it (103 IU/mL [IQR 20–656] vs. 458 IU/mL [IQR 101–3316]), although this difference was not statistically significant (*p* = 0.103). This secondary, post hoc analysis is hypothesis-generating and should be interpreted cautiously because antiviral treatment status and HBeAg status were unavailable.

The distribution of both indices against liver stiffness, and the location of the discordant classifications, are shown in Figure 3.

In the sensitivity analysis, including all 70 patients with an elastography result irrespective of the quality criterion, APRI above 0.5 reached a sensitivity of 53.3% and a specificity of 69.1% for significant fibrosis (15 cases). Performance was lower when examinations that did not meet the quality criterion were retained. This finding should be interpreted cautiously because the inclusion of lower-quality examinations may affect the observed agreement between the indices and transient elastography.

## 4. Discussion

In a cohort of patients with chronic hepatitis B from a hyperendemic Andean focus, APRI and FIB-4 showed moderate agreement with transient elastography, relatively high negative predictive values, low positive predictive values, and no detectable difference between them.

The most relevant finding is the contrast between the two decision levels. At the 0.5 threshold adopted by WHO in 2024, APRI identified 61.5% of patients classified as having significant fibrosis by elastography, against a pooled sensitivity of 72.9% in the meta-analysis informing those guidelines, which used biopsy as reference standard [4]. The confidence interval around our estimate (31.6–86.1) includes the pooled sensitivity of 72.9%, and an exact binomial test against that point estimate yielded *p* = 0.36. This comparison is descriptive and does not establish equivalence between the estimates or permit attribution of their differences to the reference standard. A population cohort using elastography as comparator reported 23.0% for the same cut-off [6]. These estimates come from populations differing in disease spectrum, design and reference standard, and the differences between them cannot be attributed to the reference standard alone. Discrimination, by contrast, was in the expected range: areas under the curve of 0.844 for APRI and 0.808 for FIB-4 at the significant-fibrosis level, against pooled values of 0.76 and 0.75 reported for chronic hepatitis B [17]. Table 5 sets the sensitivity estimates side by side together with the design features that separate them.

**Table 5:** Sensitivity of APRI for significant fibrosis reported against different reference standards. The estimates are presented descriptively and are not directly comparable because the studies differ in reference standard, design and clinical spectrum. The observed differences cannot be attributed to the reference standard alone, and the estimates should not be used to rank diagnostic performance across studies.

Source	Setting and Design	Reference Standard	*n*	APRI Cut-off	Sensitivity% (95% CI)	Specificity% (95% CI)
WHO meta-analysis [4]	211 studies pooled, 61,665 patients	Liver biopsy	61,665	>0.3–0.7	72.9 (70.2–75.5)	64.7 (61.0–68.2)
Ugandan cohort [6]	Rural community screening, population-based	Transient elastography > 7 kPa	63	>0.5	23.0 (not reported)	not reported
This study	Referral cohort within an elimination programme	Transient elastography > 7.0 kPa	60	>0.5	61.5 (31.6–86.1)	74.5 (59.7–86.1)

CI, confidence interval. The Ugandan estimate is reported without a confidence interval in the source publication and rests on a comparably small number of cases; its imprecision is therefore at least as large as that of the present study. Community screening and referral cohorts differ in fibrosis spectrum, which independently affects sensitivity.

Studies that, like ours, adopt elastography rather than biopsy as the comparator remain few, and they agree less on the magnitude of performance than on its shape. In 267 patients in southern Iran, APRI reached an area of 0.852 against FibroScan and outperformed both FIB-4 and the AST/ALT ratio [18]; in 247 patients in Alexandria, FIB-4 above 0.88 achieved a sensitivity of 65.4% and a specificity of 60.0% against vibration-controlled transient elastography, and the authors concluded that misclassification was frequent enough to require an additional test [19]; in 202 Thai patients, the two indices reached areas of 0.824 and 0.780 with no significant difference between them [20]; in 520 patients in India, the areas were 0.753 for significant fibrosis and 0.851 for cirrhosis [21]; and in a mixed hepatitis B and C cohort, the indices performed acceptably for cirrhosis but not for low-grade fibrosis [22]. A cohort of 460 patients with HIV/HBV co-infection reported higher concordance still, although antiretroviral therapy alters the determinants of both indices and limits its transferability to our population [23]. Read together, these results place APRI and FIB-4 consistently below elastography, with the gap widest at the lower fibrosis levels—which is precisely where the WHO treatment threshold now sits. A prospective study comparing transient elastography, point shear-wave elastography and both indices against biopsy found APRI the least accurate of the four methods, and the M and XL probes comparable to one another [24].

The likelihood ratios separate what these indices can and cannot do. For significant fibrosis, a positive result does shift the probability upward, but modestly and with wide uncertainty: APRI above 0.5 gives a positive likelihood ratio of 2.41 (1.26–4.62), and FIB-4 above 1.45 gives 1 of 2.63 (1.22–5.69). Ruling out is weaker still—negative likelihood ratios of 0.52 (0.25–1.05) and 0.58 (0.32–1.06), with intervals that touch unity—and negative predictive values of 87.5% and 85.4% mean that roughly one patient in seven or eight with a negative index remained elastography-positive. Neither index is therefore a reliable stand-alone rule-out test at the threshold that governs treatment eligibility. At the cirrhosis level, the position is different and favorable: positive likelihood ratios of 9.46 (3.30–27.11) for APRI above 1.0, 18.93 (4.49–79.73) for APRI above 1.5 and 35.71 (4.85–262.86) for FIB-4 above 3.25, all with intervals excluding unity, together with negative predictive values above 96%. The defensible reading is that these indices identify cirrhosis well enough to act on and are only weakly informative at the threshold that decides who starts treatment—which is the clinically consequential half of the result. A referral rule of this kind has been formalized elsewhere: in 2014 Chinese patients with chronic hepatitis B, an APRI below 0.35 or above 0.6 classified fibrosis correctly with an accuracy above 80% and would have spared elastography in roughly two-thirds of the cohort [25]. Within a WHO-aligned two-tiered pathway, APRI may serve as an accessible first-line assessment at primary healthcare facilities because it requires only AST and platelet counts, while transient elastography can provide further assessment at regional or mobile services. However, in this cohort, a negative APRI result did not reliably rule out significant fibrosis. Therefore, a negative result should not close the episode but should prompt scheduled reassessment of transaminases and platelet counts, with referral if clinical or laboratory deterioration occurs.

Regarding the comparison between indices, DeLong’s test detected no statistically significant difference between APRI and FIB-4 at either the significant-fibrosis level (*p* = 0.47) or the cirrhosis level (*p* = 0.53). The differences in AUC were small in this sample, but the limited number of outcome-positive cases (13 for significant fibrosis and 7 for cirrhosis) restricts the precision of the comparison. These results do not establish equivalence, and clinically important differences between the indices cannot be excluded. Accordingly, the data do not support claiming the superiority of either index. A similar absence of a statistically significant difference has been reported in another study using elastography as the comparator (AUCs of 0.824 and 0.780; *p* = 0.33) [20]. The choice may rest on operational grounds—APRI requires only AST and platelets, and FIB-4 adds ALT and age—rather than on a differential performance we could not demonstrate.

The thresholds applied during the Plan and those adopted by WHO in 2024 differed in this cohort by a single reclassified patient, indicating that the operational criteria used in Huanta in 2019 were already, in practice, close to those the Organization would recommend five years later.

The weak inverse associations between viral load and some fibrosis measures should be interpreted cautiously. Antiviral treatment status and HBeAg status were unavailable, and the timing of viral-load measurement relative to the other assessments could not be verified. These factors limit interpretation, and the findings should be considered hypothesis-generating rather than confirmatory. It could equally reflect antiviral treatment in those with more advanced disease; the database does not record treatment status, so the two explanations are indistinguishable. A further caveat concerns the shape of the association rather than its direction. In 5880 non-cirrhotic treatment-naive patients, the relation between viral load and fibrosis was not monotonic but parabolic, peaking at intermediate loads of around 6 log10 IU/mL [26]; a rank correlation, which assumes monotonicity, would misdescribe such a relation. We therefore tested for curvature by adding a quadratic term in log10 viral load among the 66 patients with a detectable load: it was not significant (F = 1.15, *p* = 0.29), and stiffness declined monotonically across load tertiles (median 5.95, 4.75 and 4.40 kPa). Our data are nonetheless a weak test of that hypothesis, since only four patients exceeded 5 log10 IU/mL and the range in which the reported maximum lies is barely represented. The finding remains hypothesis-generating.

Among the strengths, this work provides initial performance data, to our knowledge, for APRI and FIB-4 in an Andean population with chronic hepatitis B, in a region whose burden is documented [10]; it uses as comparator an elastography device exceptionally available in a rural referral hospital; it accompanies its estimates with exact intervals and subjects the between-index comparison to a formal statistical test; and it reports according to STARD 2015 with available analysis code and results independently reproduced in two computing environments. A further strength is that the quality criterion was verified rather than assumed: examinations with an IQR/M above 30% showed no association between the serological indices and liver stiffness (ρ = −0.26 for APRI, *p* = 0.47), whereas those meeting the criterion did (ρ = +0.57, *p* < 0.001; difference between correlations *p* = 0.02). Their exclusion is therefore supported by the behavior of the data and not only by the protocol. A published precedent points the same way: in the prospective cohort cited above, the single patient with stage F1 fibrosis whose stiffness exceeded the cirrhosis threshold had an IQR/median ratio of 87% [15]—a value close to the two most extreme examinations excluded here.

The limitation this study documents is already being addressed by tests that Huanta does not have. Fibrosis indices augmented with metabolomic markers and machine learning outperform both FIB-4 and elastography across fibrosis stages [27], and elastography-derived composite indices outperform APRI and FIB-4 in chronic hepatitis B [28]. None of these is deployable in a rural Andean network in the near term, which is why the performance of the two indices that are deployable there remains worth measuring.

This study has some limitations. The main limitation is the absence of liver biopsy, which was not available in the study setting. Transient elastography was therefore used as the reference standard, and the reported estimates should be interpreted as agreement with elastography rather than accuracy against histological fibrosis. Because the errors of the index tests and of the reference standard may be conditionally dependent, we make no claim about the direction of the resulting bias. Liver stiffness may also increase because of necroinflammatory activity independently of fibrosis. In this cohort, alanine aminotransferase correlated with liver stiffness (ρ = 0.487; *p* < 0.001), as did aspartate aminotransferase, and 14 of 66 patients exceeded twice the upper limit of normal for ALT. The same association has been reported against biopsy in chronic hepatitis B, where liver stiffness correlated with METAVIR activity grade and both aminotransferases [15]. Eleven examinations with an IQR/M ratio above 30% were excluded under the protocol criterion; in that subgroup, the indices showed no association with stiffness, and the full set is reported as a sensitivity analysis. Viral genotype was not determined, although genotype F is prevalent in the region and has been associated with a distinct clinical course [9]. Consequently, the potential influence of genotype on the findings could not be assessed. The exploratory age-stratified analysis also suggests that FIB-4 classification may be influenced by age in this cohort, consistent with its limited validation in people younger than 35 years [2]. However, the small number of fibrosis cases in each age stratum precludes firm conclusions. Under the program protocol, transient elastography and blood sampling were intended to take place during the same session, with a planned interval of zero days. However, individual dates were not recorded, so the actual interval could not be verified, and deviations from the intended timing cannot be excluded. Consequently, changes in antiviral treatment, viral activity or hepatic inflammation between assessments, if they occurred, could have affected the comparability of the measurements. The relatively small sample size, particularly the limited number of patients with significant fibrosis and cirrhosis, resulted in wide confidence intervals and limited the precision of the diagnostic estimates. With 13 cases of significant fibrosis and 7 of cirrhosis, larger multicenter prospective validation is required before these estimates are used to set local policy. Other factors that may influence the performance of non-invasive fibrosis indices, including hepatic steatosis, were not systematically characterized in the available records [12,13]. Body mass index was recorded, and 26 of 73 patients (35.6%) were at or above 25 kg/m^2^, but hepatic ultrasound and lipid profile were not, so steatosis could neither be identified nor adjusted for. Finally, the retrospective, single-center design may limit generalizability to other populations and healthcare settings, as the clinical spectrum and prevalence of fibrosis can differ substantially between community and referral populations [29]. Nevertheless, the study reflects the real-world population evaluated within the Hepatitis B Elimination Plan in a rural Andean setting where access to non-invasive fibrosis assessment is limited.

## 5. Conclusions

In this Andean population with chronic hepatitis B, APRI and FIB-4 showed moderate agreement with transient elastography. No statistically significant difference in discrimination was detected between them; however, with only 13 cases of significant fibrosis and 7 cases of cirrhosis, equivalence has not been established, and clinically important differences cannot be excluded. Their usefulness depended on the decision level. For cirrhosis, positive likelihood ratios between 9 and 36, with intervals excluding unity, make a value above threshold worth acting on. For significant fibrosis—the threshold that governs treatment eligibility under the 2024 WHO guidelines—both indices were only weakly informative, and the negative likelihood ratios did not exclude unity, so a low value cannot by itself rule out fibrosis. Where elastography is unavailable, APRI may serve as an accessible first-line assessment within a two-tiered pathway, with transient elastography used for further evaluation when indicated. However, neither index should be used alone to rule out significant fibrosis, and a negative result should trigger scheduled reassessment rather than discharge. Larger multicenter studies with a documented reference standard are needed before these estimates inform local policy.

## Figures and Tables

**Figure 1:**
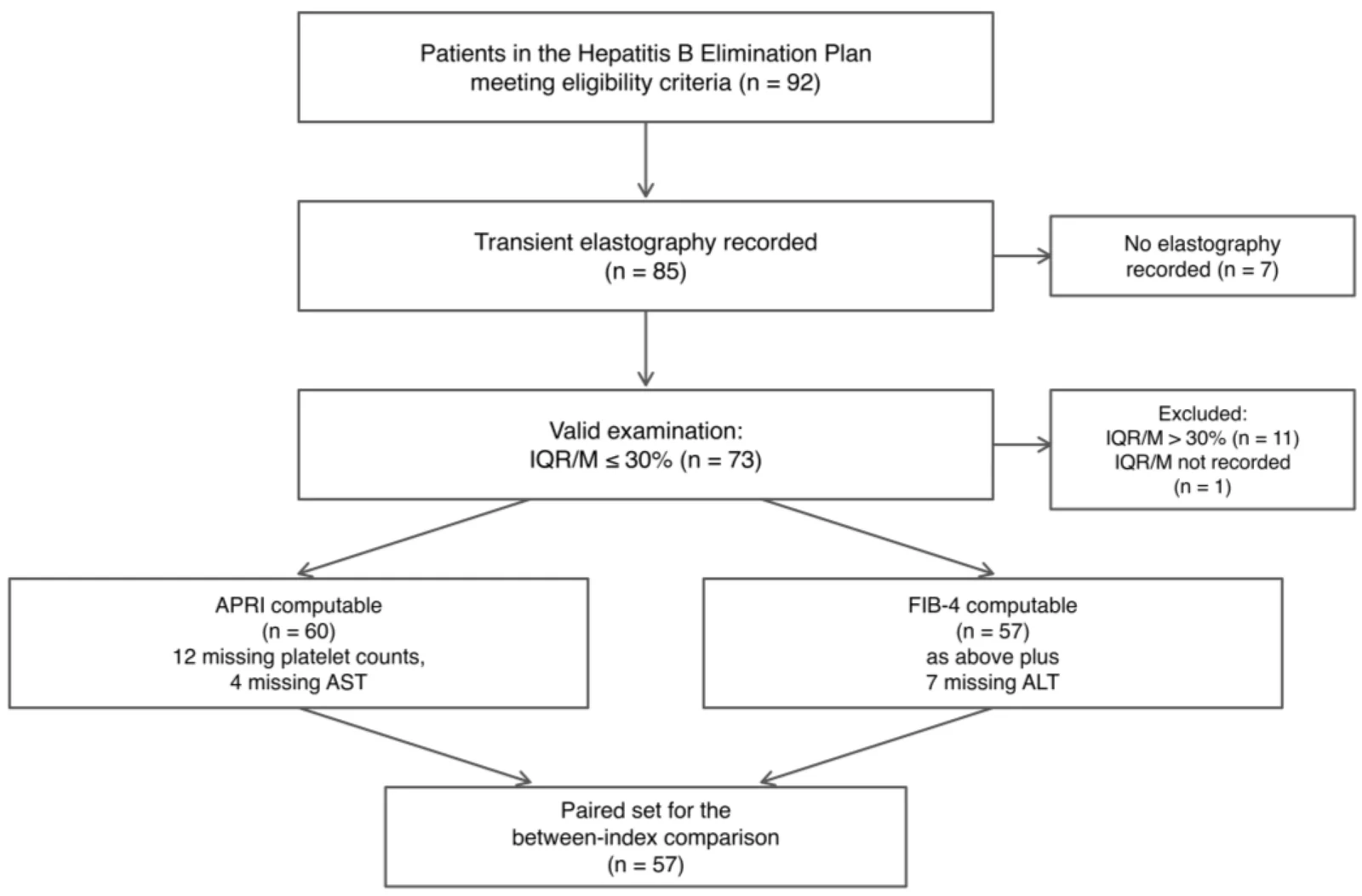
STARD flow diagram. Of 92 eligible patients, 85 had a recorded transient elastography examination, and 73 met the pre-specified quality criterion. APRI was computable in 60 and FIB-4 in 57; the paired set for the between-index comparison comprised 57 patients.

**Figure 2:**
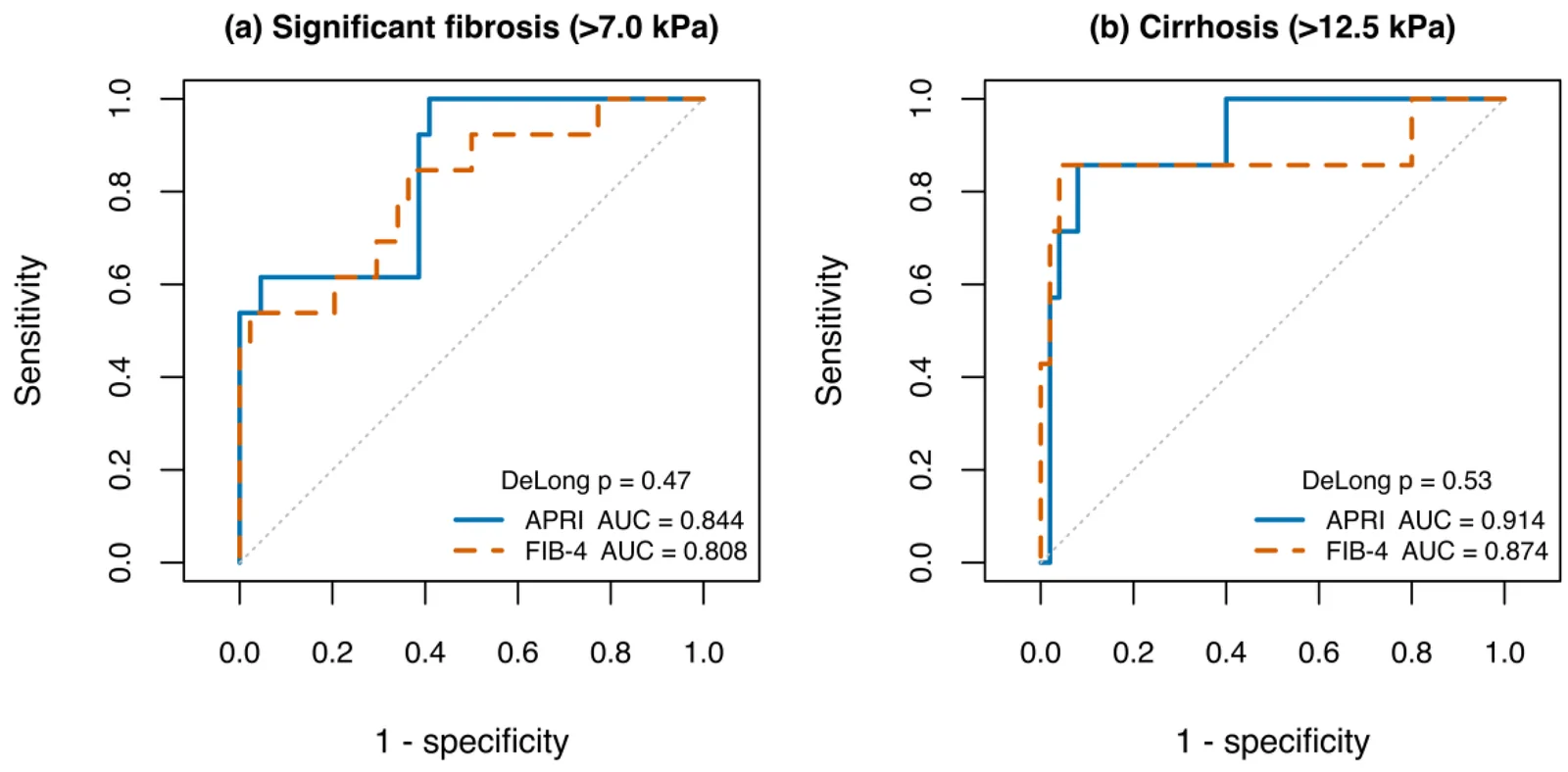
Receiver operating characteristic curves for APRI (solid, blue) and FIB-4 (dashed, orange) against transient elastography at the two primary outcome levels: (**a**) significant fibrosis (>7.0 kPa) and (**b**) cirrhosis (>12.5 kPa). Areas under the curve and the *p*-value of DeLong’s test for paired curves are shown in each panel. No statistically significant difference between indices was detected at either level. Estimated on the 57 patients with both indices and a valid elastography examination.

**Figure 3:**
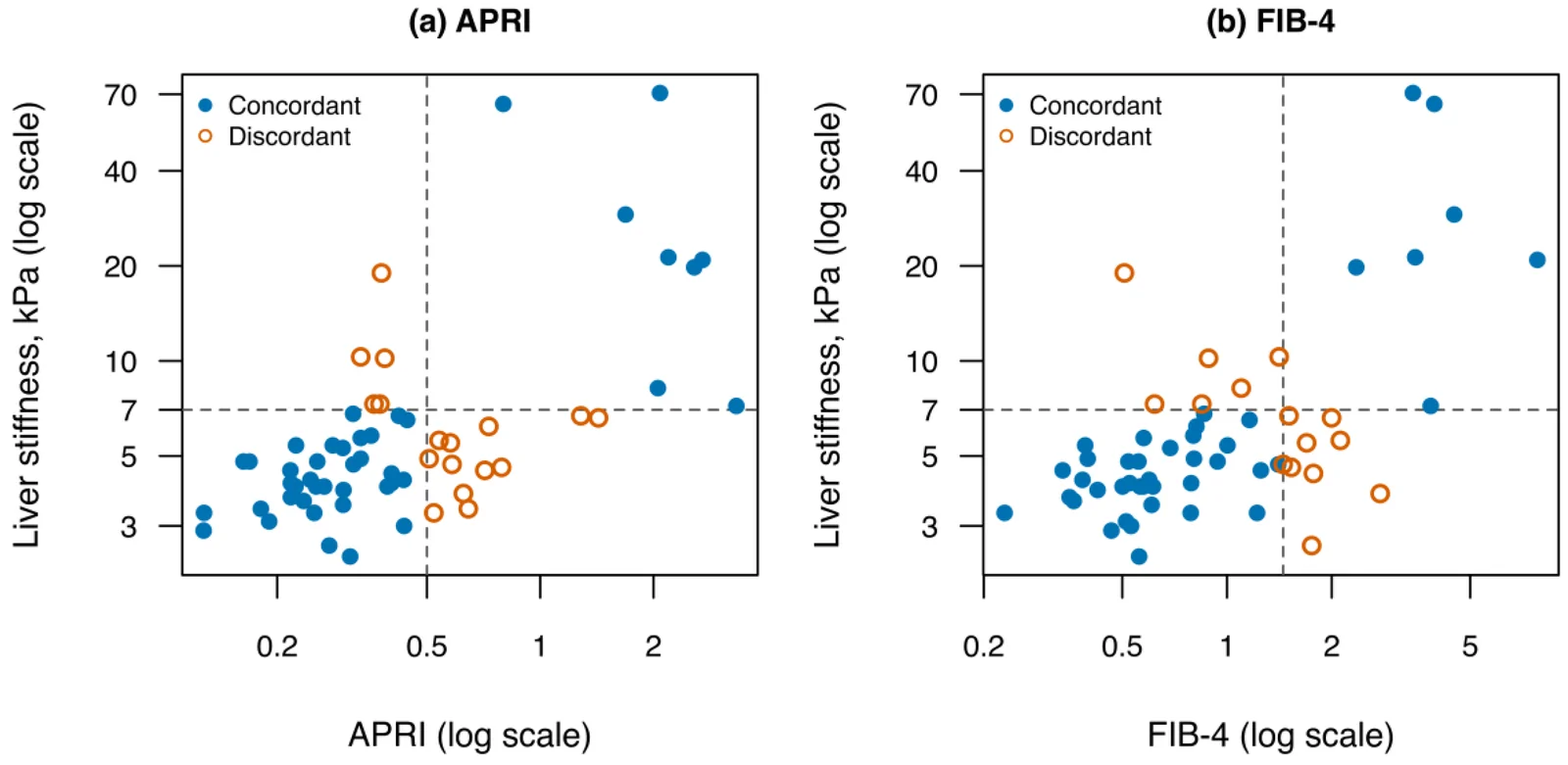
Serological indices against liver stiffness on logarithmic axes: (**a**) APRI and (**b**) FIB-4. Dashed lines mark the WHO threshold for significant fibrosis (7.0 kPa) and the index cut-off (0.5 for APRI, 1.45 for FIB-4), dividing the plane into concordance quadrants. Filled circles are concordant classifications; open circles are discordant. The lower-right quadrant contains index-negative/transient-elastography-positive discordances—patients classified as having significant fibrosis by elastography whom the index would not have referred. These are not labeled false negatives, because the reference standard is itself imperfect.

**Table 1:** Characteristics of the participants.

Characteristic	Whole Cohort (*n* = 92)	Analyzed (*n* = 57)
Age, years—median (IQR *)	36 (28–46)	33 (27–50)
Male sex, *n* (%)	46 (50.0)	32 (56.1)
Body mass index, kg/m^2^—median (IQR *)	23.4 (20.3–27.0)	23.8 (21.5–27.3)
Body mass index ≥ 28 kg/m^2^, *n* (%)	15 (16.3)	9 (15.8)
HBV viral load, IU/mL—median (IQR *)	330 (31–3438)	455 (48–2368)
Reported risk factors, *n* (%)		
Dental care in local practices	77 (83.7)	—
Family member with hepatitis B	45 (48.9)	—
Previous surgery	26 (28.3)	—
Needle-stick accident	20 (21.7)	—
Any infectious comorbidity	38 (41.3)	—
Sexually transmitted infection	6 (6.5)	—
Blood transfusion	6 (6.5)	—
Tattoos	4 (4.3)	—

* IQR, interquartile range. Risk factors were recorded for the whole cohort only. The infectious comorbidities recorded were typhoid fever, malaria, sexually transmitted infection, dengue and leishmaniasis.

**Table 2:** Distribution of liver fibrosis by transient elastography, using the 2024 WHO thresholds (*n* = 73).

METAVIR-Equivalent Band	*n* (%)	Grouped Outcome	*n*/73 (%)	95% CI *
F0/F1 (≤7.0 kPa)	56 (76.7)	—	—	—
F2–F3 (>7.0 to ≤12.5 kPa)	8 (11.0)	Significant fibrosis (>7.0 kPa)	17 (23.3)	14.2–34.6
F4 (>12.5 kPa)	9 (12.3)	Cirrhosis (>12.5 kPa)	9 (12.3)	5.8–22.1
Total	73 (100)	—	—	—

* CI, confidence interval (exact Clopper–Pearson).

**Table 3:** Diagnostic properties against transient elastography *.

Test	Target	*n*	Cases	Sn% (95% CI)	Sp% (95% CI)	PPV% (95% CI)	NPV% (95% CI)	LR+ (95% CI)	LR− (95% CI)
APRI > 0.5 (WHO)	≥F2	60	13	61.5 (31.6–86.1)	74.5 (59.7–86.1)	40.0 (19.1–63.9)	87.5 (73.2–95.8)	2.41 (1.26–4.62)	0.52 (0.25–1.05)
APRI > 1.5	≥F2	60	13	53.8 (25.1–80.8)	100 (92.5–100)	100 (59.0–100)	88.7 (77.0–95.7)	not estimable †	0.46 (0.26–0.83)
FIB-4 > 1.45	≥F2	57	13	53.8 (25.1–80.8)	79.5 (64.7–90.2)	43.8 (19.8–70.1)	85.4 (70.8–94.4)	2.63 (1.22–5.69)	0.58 (0.32–1.06)
APRI > 1.0 (WHO)	F4	60	7	71.4 (29.0–96.3)	92.5 (81.8–97.9)	55.6 (21.2–86.3)	96.1 (86.5–99.5)	9.46 (3.30–27.11)	0.31 (0.10–1.00)
APRI > 1.5	F4	60	7	71.4 (29.0–96.3)	96.2 (87.0–99.5)	71.4 (29.0–96.3)	96.2 (87.0–99.5)	18.93 (4.49–79.73)	0.30 (0.09–0.96)
FIB-4 > 3.25	F4	57	7	71.4 (29.0–96.3)	98.0 (89.4–99.9)	83.3 (35.9–99.6)	96.1 (86.5–99.5)	35.71 (4.85–262.86)	0.29 (0.09–0.94)

* Sn, sensitivity; Sp, specificity; PPV and NPV, positive and negative predictive value; LR, likelihood ratio. Estimates rest on 13 cases of significant fibrosis and 7 of cirrhosis, and the intervals are correspondingly wide. † The positive likelihood ratio is not estimable because specificity reached 100%. Confidence intervals for likelihood ratios were obtained by the log method. Predictive values depend on the prevalence and clinical spectrum of this referral cohort and should not be transferred unchanged to a screened population.

**Table 4:** Areas under the curve and paired comparison *.

Level	AUC APRI (95% CI)	AUC FIB-4 (95% CI)	Difference	*p* (DeLong)
Significant fibrosis (>7.0 kPa)	0.844 (0.715–0.953)	0.808 (0.644–0.937)	+0.037	0.47
Cirrhosis (>12.5 kPa)	0.914 (0.788–1.000)	0.874 (0.591–1.000)	+0.040	0.53

* Estimated on the 57 patients with both indices and a valid elastography examination. Bootstrap intervals from 2000 replicates.

## Data Availability

The analysis dataset is fully de-identified: it contains no names, identity document numbers, clinical record numbers or any variable permitting re-identification. The dataset, the analysis code and the data preparation pipeline are available from the corresponding author upon reasonable request.

## Supplementary Materials

The following supporting information can be downloaded at https://www.mdpi.com/article/10.3390/medsci14060670/s1, Supplementary File S1: Statistical analysis code (R) for APRI and FIB-4 evaluation.

## Abbreviations

The following abbreviations are used in this manuscript:

ALT	Alanine aminotransferase
APRI	Aspartate aminotransferase to platelet ratio index
AST	Aspartate aminotransferase
AUC	Area under the curve
CI	Confidence interval
FIB-4	Fibrosis-4 index
HBV	Hepatitis B virus
IQR	Interquartile range
LR	Likelihood ratio
NPV	Negative predictive value
PPV	Positive predictive value
ROC	Receiver operating characteristic
STARD	Standards for Reporting of Diagnostic Accuracy Studies
ULN	Upper limit of normal
WHO	World Health Organization
